# Cerebral Venous Thrombosis: Pathophysiologic Insights, Clinical Evaluation Tools, and Novel Therapeutic Strategies

**DOI:** 10.3390/diagnostics16091308

**Published:** 2026-04-27

**Authors:** Min Li, Qiqi Cui, Xiaogang Gao, Xuefan Yao, Ran Meng, Xunming Ji, Juexian Song

**Affiliations:** 1 Department of Neurology, Xuanwu Hospital Capital Medical University, Beijing 100053, China; limin@xwhosp.org (M.L.); cuiqiqi2002@163.com (Q.C.); kwithna.y@gmail.com (X.Y.); ranmeng2011@126.com (R.M.); 2 Department of Medicine, Tianjin Huanhu Hospital, Tianjin 300350, China; hyyneike@163.com; 3 Department of Neurosurgery, Xuanwu Hospital Capital Medical University, Beijing 100053, China

**Keywords:** cerebral venous thrombosis, pathophysiology, diagnosis, evaluation tools, novel therapy

## Abstract

Cerebral venous thrombosis (CVT) is a rare but potentially life-threatening subtype of stroke, characterized by thrombus formation within the dural venous sinuses and cerebral veins. Recent advances have deepened our understanding of CVT pathophysiology, highlighting a multifactorial process that encompasses thrombus initiation, subsequent thrombus propagation, venous hypertension with blood–brain barrier disruption, and secondary parenchymal brain injury. Comprehensive clinical assessment, including diagnosis and differential diagnosis, disease severity scores, imaging-based metrics, and prognostic scoring systems, enables accurate evaluation and risk stratification. Emerging therapeutic strategies, including direct oral anticoagulants, corticosteroids for selected patients, natural-origin agents, immunomodulatory therapy, endovascular treatment, optic nerve sheath fenestration, and neuromodulation, provide novel and alternative options for the management of CVT. This review provides a comprehensive overview of CVT pathophysiology, clinical assessment tools, and novel therapeutic strategies to guide clinical decision-making and inform future research.

## 1. Introduction

Cerebral venous thrombosis (CVT) refers to the formation of thrombi within the intracranial venous system, involving the dural venous sinuses and/or the cortical and deep cerebral veins. It constitutes a rare but potentially life-threatening subtype of stroke, accounting for approximately 0.5–1.0% of all stroke admissions [1]. The global male-to-female ratio among incident cases is approximately 1:2.2, whereas in Asia the corresponding ratio is about 1:1.7 [2]. Female patients most commonly develop the disease in the context of estrogen–progestin-related exposures and the contribution of inherited thrombophilia-associated genetic variants. In contrast, disease onset in male patients appears to be driven predominantly by acquired prothrombotic states, including malignancy and systemic infections; notably, several non-sex-specific risk factors tend to be overrepresented in male cohorts [3,4]. Recurrence after CVT appears to be approximately 1.5–3% at 1 year, about 12% at 5 years, and roughly 18% at 10 years [5,6].

Traditional reviews of CVT pathophysiology have primarily focused on venous stasis and elevated venous pressure, emphasizing their consequences such as blood–brain barrier (BBB) disruption, vasogenic edema, and venous infarction, as well as impaired cerebrospinal fluid (CSF) absorption leading to intracranial hypertension. However, this perspective provides an incomplete picture, as it does not fully capture the processes underlying thrombus initiation, propagation, BBB disruption, and the subsequent complex cascade of secondary parenchymal injury. This review offers a more integrative understanding of CVT pathophysiology.

Clinical manifestations of CVT are often heterogeneous and non-specific, which can lead to misdiagnosis or delayed diagnosis. In this review, we summarize current approaches to the diagnosis and differential diagnosis of CVT. Although several tools exist for clinical evaluation of CVT, including disease severity scores, imaging-based scores for venous occlusion, venous recanalization, and collateral circulation, and prognostic scores, the current literature lacks a systematic review and comparative analysis of these scales. Consequently, clinicians have limited guidance on selecting the most appropriate tool for specific clinical scenarios. This review provides a comprehensive summary of these evaluation tools to guide their clinical application [7].

In addition, insights into CVT pathophysiology have driven the development of emerging therapies beyond standard anticoagulation, including direct oral anticoagulants, corticosteroids, natural-origin agents, immunomodulatory therapy, endovascular treatment, optic nerve sheath fenestration, and neuromodulatory approaches such as rhythmic deep-brain magnetic stimulation. We discuss these evolving strategies and their therapeutic effects, offering insights for clinical decision-making and future research directions.

## 2. Literature Search Strategy

This narrative review was conducted through a structured literature search to identify relevant studies on CVT, with particular emphasis on pathophysiological mechanisms, clinical evaluation tools, and emerging therapeutic strategies. Electronic databases including PubMed, Web of Science, and Scopus were searched for articles published from January 1966 to January 2026. The search strategy combined Medical Subject Headings (MeSH) terms and free-text keywords, including “cerebral venous thrombosis”, “cerebral venous sinus thrombosis”, “CVT”, “pathophysiology”, “diagnosis”, “clinical scale”, “severity score”, “prognostic score”, “imaging score”, “treatment”, and “therapy”. Studies were included if they (1) investigated pathophysiology, diagnostic approaches, clinical evaluation scales, or treatment strategies for CVT, and (2) were original research articles, clinical trials, cohort studies, or high-quality reviews published in English. Conference abstracts without full text and studies unrelated to CVT were excluded.

## 3. Pathophysiological Mechanisms of CVT

### 3.1. Stage I: Thrombus Initiation

**Figure 1:**
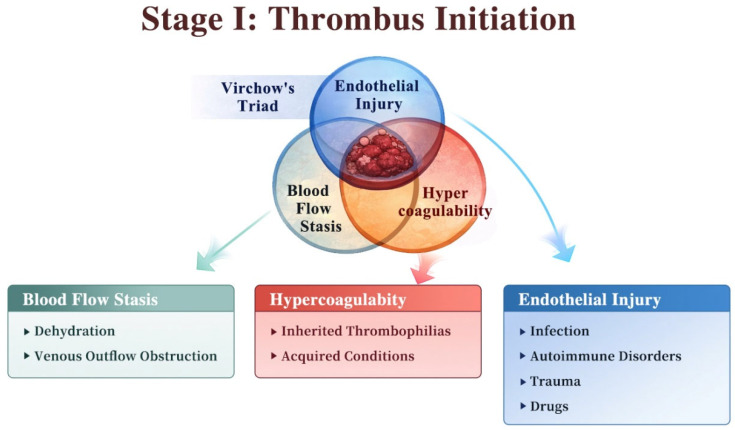
The initiation of CVT is driven by Virchow’s triad, including blood flow stasis, endothelial injury, and hypercoagulability, which often interact to promote thrombus formation in the cerebral veins and dural sinuses.

As illustrated in Figure 1, CVT results from thrombus formation within the cerebral veins or dural venous sinuses and is initiated by Virchow’s triad—blood flow stasis, endothelial injury, and hypercoagulability—which often coexist and interact under diverse clinical conditions [8]. The cerebral venous system lacks valves and operates under low-pressure, low-velocity conditions, rendering it intrinsically prone to blood flow stasis. This vulnerability is further increased by dehydration and venous outflow obstruction such as venous narrowing or maldevelopment. These factors can markedly reduce venous flow velocity, enhancing erythrocyte and platelet margination along the vessel wall and triggering activation of the coagulation cascade. In parallel, infection (e.g., sinusitis, otitis), autoimmune disorders (e.g., systemic lupus erythematosus, Behçet’s disease), mechanical trauma, and certain drugs (e.g., chemotherapeutic agents) can directly damage the venous sinus endothelium [9]. This endothelial injury results in upregulation of tissue factor and adhesion molecules, as well as increased release of von Willebrand factor, thereby facilitating platelet adhesion and fibrin deposition [10]. Furthermore, inherited thrombophilias (e.g., antithrombin III deficiency, protein C/S deficiency, factor V Leiden mutation) and acquired conditions (pregnancy, puerperium, oral contraceptive use, malignancy, antiphospholipid syndrome) create a systemic hypercoagulable state characterized by enhanced thrombin generation and suppressed endogenous fibrinolysis, thereby substantially increasing the risk of CVT [11,12].

### 3.2. Stage II: Thrombus Propagation

Once initiated, thrombus stabilization and extension are governed by neutrophil extracellular trap (NET) formation and the suppression of fibrinolysis. Platelet factor 4 (PF4) released from activated platelets induces neutrophils to release NETs composed of DNA scaffolds and granular proteins [13,14]. NETs promote thrombogenicity by providing a structural framework for fibrin and platelets and binding to additional PF4 [15]. NET-bound PF4 not only stabilizes thrombus structure but also amplifies platelet activation, creating a positive feedback loop that further reinforces thrombus formation.

**Figure 2:**
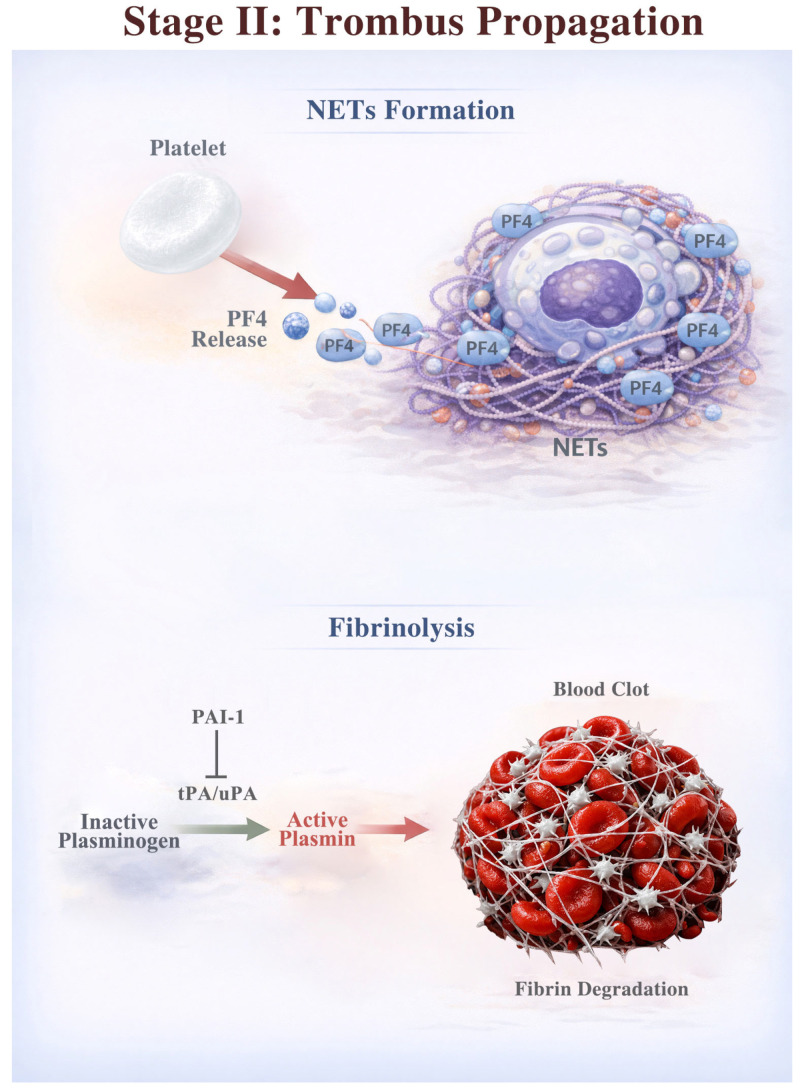
Mechanisms of thrombus propagation in CVT. Thrombus propagation is driven by NET formation and suppressed fibrinolysis. Platelet-derived PF4 induces NET release, providing a scaffold for fibrin and platelets and amplifying platelet activation in a feed-forward loop that stabilizes and enlarges the thrombus. Concurrently, increased PAI-1 inhibits tPA/uPA-mediated plasmin generation, reducing fibrinolysis and delaying recanalization.

Moreover, the fibrinolytic system is suppressed by plasminogen activator inhibitors (PAIs) in CVT [16]. Among the two isoforms, PAI-1 is generally produced in activated endothelial cells, platelets, and leukocytes, while PAI-2 is locally produced by monocytes and macrophages. Blood PAI-1 has been reported to be upregulated, whereas the status of PAI-2 remains unknown [17]. PAI-1 forms stable, irreversible complexes with tissue-type plasminogen activator (tPA) and urokinase-type plasminogen activator (uPA), thereby preventing the conversion of plasminogen into active plasmin. This mechanism makes venous thrombi more resistant to endogenous fibrinolysis and delays recanalization. The mechanisms underlying thrombus propagation are illustrated in Figure 2.

### 3.3. Stage III: Venous Hypertension and BBB Disruption

**Figure 3:**
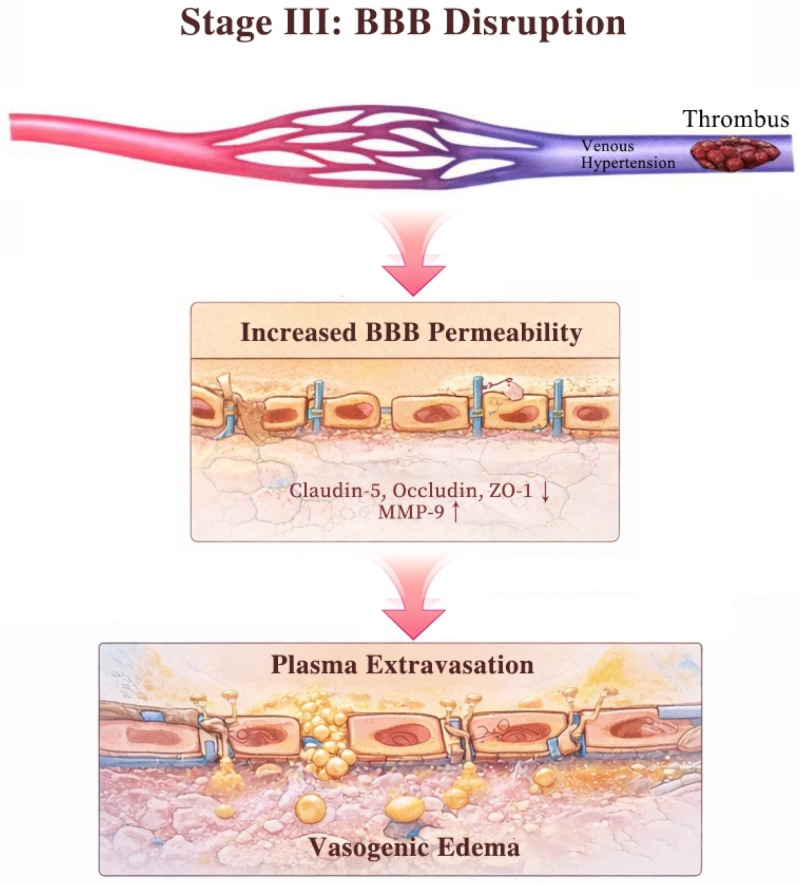
Pathophysiological mechanisms of venous hypertension-induced BBB disruption in CVT. Venous thrombosis induces venous hypertension and elevated capillary hydrostatic pressure, leading to down-regulation of tight junction proteins and BBB disruption, resulting in plasma extravasation and vasogenic edema. ↓ indicate down-regulation; ↑ indicate up-regulation.

As shown in Figure 3, occlusion of cerebral veins or dural sinuses elevates upstream venous pressure, producing venous hypertension. The backward transmission of venous pressure to the microcirculation increases capillary hydrostatic pressure, leading to down-regulation of tight junction proteins, including claudin-5, occludin, and zonula occludens-1 (ZO-1), thereby increasing BBB permeability [18]. In parallel, locally released matrix metalloproteinase-9 (MMP-9) mediates proteolytic degradation of basement membrane components and tight junction proteins, amplifying BBB breakdown [19]. This disruption facilitates plasma extravasation into the interstitial space and manifests radiologically and clinically as vasogenic edema. Concomitantly, thrombosis of major dural sinuses impairs CSF absorption via the arachnoid granulations, representing a primary driver of raised intracranial pressure, papilledema, and headache.

### 3.4. Stage IV: Parenchymal Brain Injury

**Figure 4:**
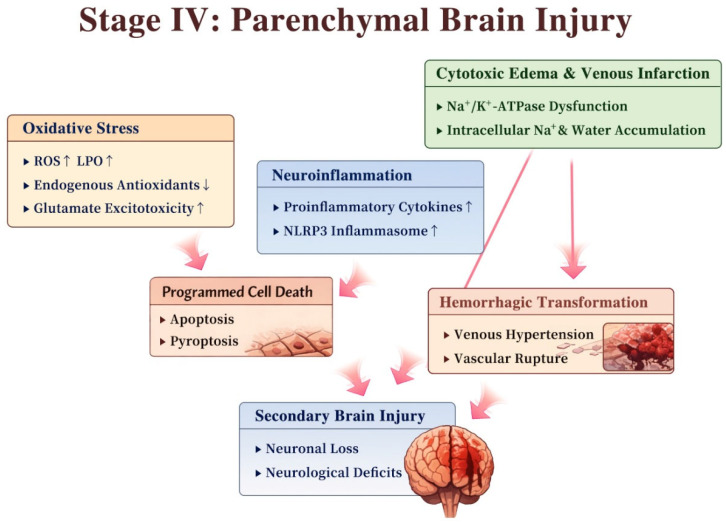
Secondary parenchymal injury following CVT. Sustained venous congestion and BBB disruption trigger a cascade of secondary parenchymal injury, including venous infarction, hemorrhagic transformation, neuroinflammation, oxidative stress, and multiple forms of programmed cell death. ↓ indicate down-regulation; ↑ indicate up-regulation.

Following sustained venous congestion and BBB disruption, CVT gives rise to a complex cascade of secondary parenchymal injury, characterized by venous infarction, neuroinflammation, oxidative stress, and convergent forms of programmed cell death, as illustrated in Figure 4.

Prolonged capillary hypoperfusion and tissue hypoxia impair Na^+^/K^+^-ATPase activity, leading to intracellular sodium and water accumulation in neurons and glial cells and thereby promoting cytotoxic edema and venous infarction. Consistently, animal models of severe CVT have demonstrated parasinus infarction, as evidenced by triphenyltetrazolium chloride staining, providing direct histopathological support for ischemic venous infarction [20]. Furthermore, persistently elevated venous pressure can also cause rupture of structurally vulnerable vessels, resulting in hemorrhagic transformation, which occurs more frequently in CVT than in arterial stroke [21].

Inflammation and oxidative stress represent key amplifying mechanisms in secondary brain injury following CVT. Experimental CVT models have demonstrated prominent accumulation of neutrophils and monocyte–macrophages in affected regions, accompanied by increased expression of proinflammatory cytokines and chemokines, including tumor necrosis factor-α, interleukin (IL)-1β, and IL-6 [22]. At the molecular level, activation of the NOD-like receptor family pyrin domain-containing 3 (NLRP3) inflammasome leads to caspase-1 activation, maturation of IL-1β and IL-18, and gasdermin-D-mediated pyroptosis, thereby promoting neuroinflammation and neuronal injury [23].

Concurrently, oxidative stress further exacerbates neuronal damage. Experimental models have demonstrated reduced activities of endogenous antioxidant defenses, including manganese superoxide dismutase, catalase, glutathione peroxidase, and glutathione, together with increased levels of reactive oxygen species (ROS) and lipid peroxidation (LPO), indicating a pronounced redox imbalance [24]. In parallel, excessive glutamate signaling and mitochondrial dysfunction contribute to reactive oxygen species generation, ultimately activating the intrinsic apoptotic pathway characterized by an increased Bax/Bcl-2 ratio and subsequent caspase-3 activation [24,25,26].

These pathways interact in a vicious cycle to amplify secondary parenchymal damage and worsen neurological outcomes following CVT. Nissl staining and NeuN immunostaining further revealed a significant reduction in neuronal density in the parasinus cortex, indicating overt neuronal loss. Clinical studies have also reported elevated serum levels of neuron-specific enolase (NSE) in patients with CVT, which reflect neuronal damage and correlate with infarct burden, hemorrhagic transformation, and disease severity [27].

## 4. Clinical Evaluation Tools

### 4.1. Diagnosis and Differential Diagnosis

The diagnosis of CVT relies on a combination of clinical suspicion and neuroimaging confirmation. Digital subtraction angiography (DSA) has historically been regarded as the reference standard for the diagnosis of CVT because of its high spatial and temporal resolution in depicting venous sinus occlusion, delayed venous drainage, and collateral circulation. However, owing to its invasive nature, the routine diagnosis of CVT is currently based primarily on noninvasive imaging techniques, particularly magnetic resonance imaging (MRI) combined with magnetic resonance venography (MRV) and computed tomography venography (CTV) [28]. In clinical practice, MRI/MRV is generally considered the first-line imaging modality, whereas DSA is reserved for equivocal cases or when endovascular intervention is contemplated. The diagnosis is established when imaging demonstrates thrombosis of the cerebral veins or dural sinuses in conjunction with compatible clinical findings [29].

Differentiating CVT from non-thrombotic cerebral venous sinus stenosis (CVSS) remains a diagnostic challenge, particularly in patients presenting with intracranial hypertension or venous filling defects. CVSS, which is commonly associated with idiopathic intracranial hypertension, typically manifests as smooth, tapered narrowing of the transverse or sigmoid sinus. In contrast, CVT is characterized by intraluminal thrombus formation, manifested as abrupt or irregular intraluminal narrowing or occlusion, and may be accompanied by parenchymal venous infarction or hemorrhage [30]. In addition, imaging markers of intracranial hypertension, including increased peri-optic cerebrospinal fluid pressure, optic nerve head protrusion, scleral flattening, and empty sella, are observed more frequently in patients with CVSS-associated intracranial hypertension than in those with CVT-associated intracranial hypertension [31].

Conventional MRV and CTV, which rely on flow-related signal alterations, are limited in distinguishing CVT from CVSS mimics [32]. In this context, contrast-enhanced black-blood MRI enables direct visualization of intraluminal thrombus through effective suppression of blood flow signal, thereby overcoming the inherent limitations of flow-dependent techniques [33]. This method has emerged as a highly sensitive modality that may provide superior diagnostic accuracy, particularly in cases with slow flow, partial recanalization, isolated cortical vein thrombosis, or equivocal findings on routine venography. Accordingly, black-blood MRI represents an important complementary tool for the diagnosis of cerebral venous thrombosis and may substantially improve diagnostic confidence in challenging cases [34].

In the acute phase, blood biomarkers such as fibrinogen and D-dimer may help differentiate CVT from CVSS [35]. Moreover, CVT is associated with a more pronounced inflammatory response than CVSS, with elevated interleukin-6 levels exceeding 3.06 pg/mL, which may serve as a useful marker for distinguishing CVT from CVSS in both the acute and subacute phases [36]. The key features differentiating CVT from CVSS are summarized in Table 1.

### 4.2. Disease Severity Assessment

Severe CVT was initially defined by CVT-related complications, such as venous cerebral infarction, venous cerebral hemorrhage, subarachnoid hemorrhage, seizures, mental disorders or a decreased level of consciousness [37,38]. With increasing recognition of the heterogeneity of clinical manifestations, neurological scales have been considered more appropriate for evaluating disease severity in CVT. Traditional neurological scales, such as the Glasgow Coma Scale (GCS) and the National Institutes of Health Stroke Scale (NIHSS), were developed to assess the level of consciousness after traumatic brain injury and neurological deficits after arterial stroke, respectively [39,40]. CSF pressure can only reflect the degree of intracranial hypertension [41]. These assessments are not CVT-specific and only partially capture the clinical manifestations of CVT.

Recognizing this gap, two CVT-specific scales for evaluating disease severity were recently developed. The CVT grading scale proposed by Barboza MA et al. ranges from 0 to 13 points and is composed of parenchymal lesion size > 6 cm (3 points), bilateral Babinski signs (3 points), male sex (2 points), parenchymal hemorrhage (2 points), and the level of consciousness (3 points) [42]. Based on the total score, CVT is categorized as mild (0–2 points), moderate (3–7 points), or severe (8–13 points). However, this scale was initially designed for outcome prediction in CVT rather than for severity assessment [42]. It mainly reflects focal neurological impairment and fails to incorporate manifestations such as intracranial hypertension, cavernous sinus syndrome, or diffuse encephalopathy.

The CVT severity scale proposed by Li M et al. [43] was developed based on the NIHSS and incorporates seven additional CVT-relevant items, with higher scores indicating more severe disease. This scale was prospectively validated in a multicenter Chinese cohort. Items 1–12 (level of consciousness, gaze palsy, visual field, facial palsy, motor arm, motor leg, limb ataxia, sensory, best language, dysarthria, extinction and inattention, and epilepsy) assess focal neurological deficits while items 13–15 (Headache, tinnitus, and papilledema) evaluate intracranial hypertension. Items 15–16 (papilledema and Ophthalmoplegia) reflect cavernous sinus syndrome and items 17–18 (mental disorders and neck discomfort) capture diffuse encephalopathy. The scale demonstrated strong inter-rater reliability and showed good correlations with baseline NIHSS, GCS, and CSF pressure. A score > 10 on the CVT severity scale is considered severe, indicating poor prognosis, whereas a score ≤ 10 is considered mild, suggesting a relatively favorable outcome [43]. However, this scale still requires further international validation. The comparison between traditional neurological scales and CVT-specific severity scales is presented in Table 2.

Serum biomarkers also provide valuable insights into the severity of CVT. NSE, released from damaged neurons, has been shown to correlate with infarct volume, cerebral edema, and overall clinical severity [44]. The D-dimer-to-fibrinogen ratio (DFR), which reflects the imbalance between coagulation and fibrinolysis, is associated with disease severity in CVT [45]. In addition, emerging evidence suggests that high-sensitivity C-reactive protein (hs-CRP), IL-6, the neutrophil-to-lymphocyte ratio (NLR) during the acute and subacute stages of CVT may serve as inflammatory biomarkers reflecting disease severity in CVT [36,46]. Integrating these biomarkers with clinical and neuroimaging findings enables a multidimensional assessment of CVT severity and may help inform prognosis [46]. They offer a more precise reflection of symptom severity and can serve as sensitive indicators for monitoring disease progression. Additionally, these biomarkers may be used as stratification variables in clinical trials to ensure balanced baseline severity and to guide patient selection for interventional thrombectomy [43,47].

### 4.3. Imaging Scoring Systems

Neuroimaging offers the most direct assessment of thrombus burden, venous recanalization, and collateral circulation in CVT. Accordingly, multiple imaging-based scoring systems have been proposed to convert complex radiological findings into standardized severity metrics.

#### 4.3.1. Venous Occlusion Scores

In 2009, Zubkov AY et al. [48] proposed a 11-point cerebral venous sinus thrombosis (CVST) score. 1 point was assigned for the straight sinus, vein of Galen, each internal cerebral vein, each transverse sinus, and each sigmoid sinus. The superior sagittal sinus (SSS) was divided into three segments, with 1 point allocated to each involved third.

The total CVST score was calculated by summing the scores of each affected sinus [48,49]. A higher CVST score was associated with an increased risk of parenchymal brain lesions. However, subsequent studies have shown that the CVST score does not reliably reflect clinical severity, MRI lesion burden, or functional outcome, suggesting limited prognostic value [49].

In 2021, Wu Z et al. introduced and validated a 10-point Venous Occlusion Image Score (VOIS) to quantitatively assess cortical vein and dural sinus occlusion in patients with CVST [50]. In this scoring system, the SSS was assigned 2 points, while the inferior sagittal sinus, straight sinus, each transverse sinus, each sigmoid sinus, and each internal jugular vein were each assigned 1 point. The total VOIS was determined by the cumulative sum of scores from each affected sinus. The VOIS demonstrated excellent interobserver and inter-method agreement. Baseline VOIS values were inversely correlated with stroke severity as measured by the NIHSS and mRS, and independently predicted functional outcome. However, this scoring system does not account for the inherent bilateral asymmetry of the transverse sinuses, sigmoid sinuses, and internal jugular veins.

In 2023, Wang Z et al. proposed a CVST thrombus burden score ranging from 0 to 30 points [51]. In this scoring system, the absence of thrombus was assigned 0 points, thrombus occupying ≤50% of a venous segment was assigned 1 point, thrombus occupying >50% but <100% of the segment was assigned 2 points, and complete occlusion was assigned 3 points. To account for physiological asymmetry, the drainage weight for each side was defined as the cross-sectional area of the transverse sinus on that side relative to the total cross-sectional area of both transverse sinuses [51]. The thrombus score of the transverse sinus, sigmoid sinus, and internal jugular vein was multiplied by the side-specific drainage weight. Ultimately, thrombus scores from the superior sagittal sinus, straight sinus, torcular herophili, bilateral transverse sinuses, sigmoid sinuses, and internal jugular veins were aggregated to yield the final thrombus burden score. A CVST Thrombus Burden Score > 7.15 predicted an intracranial pressure > 250 mmH_2_O, whereas a score > 11.62 predicted an ICP > 330 mmH_2_O [51].

All the aforementioned venous occlusion scoring systems are summarized in Table 3. Both the CVST score and the VOIS are simple and easy to apply in clinical practice. In the CVST score, the SSS was divided into three segments, with 1 point assigned to each segment, whereas in the VOIS, the SSS was directly assigned 2 points. Both scores include the straight sinus, bilateral transverse sinuses, and bilateral sigmoid sinuses. In contrast, the CVST score incorporates the deep venous system, including the vein of Galen and the bilateral internal cerebral veins, whereas the VOIS includes the inferior sagittal sinus and bilateral internal jugular veins. In addition, the degree of occlusion is not considered in both scores. The bilateral transverse and sigmoid sinuses are assigned equal weights regardless of physiological dominance or hypoplasia, which may limit their ability to accurately reflect the true hemodynamic impact of venous outflow obstruction in both scores. The CVST thrombus burden score incorporates the degree of occlusion and weights the transverse sinus, sigmoid sinus, and internal jugular vein scores based on the side-specific drainage ratio. However, it requires relatively complex calculations, which may limit its clinical applicability.

#### 4.3.2. Venous Recanalization Grades

In 2010, Qureshi AI et al. proposed a grading scale to evaluate recanalization [52]. According to the Qureshi recanalization grading system, Grade I indicates partial recanalization in one or more occluded sinuses. Grade II indicates complete recanalization of at least one previously occluded sinus, while occlusion persists in other sinuses. Grade II was further subclassified into Grade IIA, defined as complete recanalization without residual flow defect, and Grade IIB, defined as complete recanalization with a non-occlusive flow defect. Grade III indicates complete recanalization of all previously occluded sinuses.

In 2020, Aguiar de Sousa D et al. proposed another grading scale for venous recanalization [53]. Grade 0 indicates persistent occlusion of all previously occluded sinuses. Grade 1 indicates the presence of persistent occlusion in at least one sinus. It is subdivided into Grade 1A, defined as a combination of persistent occlusion and partial recanalization, and Grade 1B, defined as a combination of persistent occlusion and complete recanalization. Grade 2 indicates the absence of any persistent occlusion. It is subdivided into Grade 2A, defined as partial recanalization of all thrombosed sinuses, and Grade 2B, defined as a combination of partial and complete recanalization.

Grade 2 indicates the absence of any persistent occlusion, and is subdivided into Grade 2A, defined as partial recanalization of all thrombosed sinuses, and Grade 2B, defined as a combination of partial and complete recanalization. Grade 3 indicates complete recanalization of all previously thrombosed sinuses.

The Qureshi grade and the Aguiar grade are summarized in Table 3. Compared with the Qureshi grade, the Aguiar grade provides a more refined and clinically informative assessment of venous recanalization. While the Qureshi classification primarily categorizes recanalization into broad levels of partial or complete reopening, the Aguiar de Sousa scale first distinguishes the presence or absence of persistent occlusion and further subdivides partial and complete recanalization patterns within each category. This hierarchical structure allows for the identification of mixed recanalization states across different sinuses, thereby reducing information loss associated with oversimplified categories, more clearly depicting intermediate stages of sinus recanalization, facilitating the assessment of early sinus reopening, and strengthening the correlation between imaging-based recanalization and clinical outcomes.

#### 4.3.3. Collateral Circulation Grades

In 2010, Qureshi AI et al. also proposed a grading scale to evaluate the pattern of venous collateral circulation [52]. Grade I indicates that collateral veins bypass the occluded segment and reconnect with the same sinus. Grade II indicates that collateral veins bypass the occluded segment and drain into another dural venous sinus. Grade III indicates that collateral veins bypass the occluded segment and drain into the deep venous system or the cavernous sinus.

In 2018, Sheth SA et al. proposed a venous collateral scale (VCS) to evaluate the adequacy of venous collateral compensation [54]. Grade 0 indicates no venous drainage from affected region. Grade 1 indicates cortical venous drainage from affected region without anastomosis to a patent sinus. Grade 2 indicates cortical venous drainage from affected region with anastomosis into a patent sinus.

The Qureshi grade and the VCS are summarized in Table 3. Although the Qureshi grade characterized different venous bypass routes in cerebral venous thrombosis, this classification primarily describes the anatomical patterns of venous rerouting after sinus occlusion. Its grading scheme focuses on the final drainage destination of collateral channels and is mainly intended to depict venous flow reconstruction rather than the functional adequacy of venous outflow. In contrast, the VCS directly evaluates whether cortical venous drainage from the affected region is effectively established and successfully anastomosed to a patent sinus, thereby reflecting the capacity of collateral pathways to compensate for venous outflow obstruction.

### 4.4. Prognostic Scores

Although numerous prognostic indicators have been reported in previous studies, most of these markers have limited utility when used in isolation. In particular, when different indicators yield inconsistent prognostic predictions, accurate prognostic assessment becomes challenging. Therefore, integrating multiple markers into a composite scoring system may offer greater clinical utility. Over the past two decades, several CVT-specific prognostic scores have been developed to identify patients at risk of poor outcomes.

The risk score derived from the International Study on Cerebral Vein and Dural Sinus Thrombosis (ISCVT) was first proposed in 2009 by Ferro JM et al. [55] and remains a widely used prognostic tool. The ISCVT prospectively recruited patients predominantly from Europe, South America, and North America, with smaller contributions from Australia, China, and India. Its core variables include male sex, age > 37 years, coma, mental status disturbance, intracerebral hemorrhage, and deep venous system thrombosis. The ISCVT risk score ranges from 0 to 9 points, and a cutoff value of ≥3 has been shown to retain predictive value for poor functional outcomes.

Delayed clinical progression (DCP) refers to a clinical course in which a subset of patients experiences neurological deterioration following an initially stable presentation. In 2018, Bushnaq SA et al. [56] systematically identified high-risk features associated with DCP in patients with CVT from the University of New Mexico Hospitals and proposed a prediction score to facilitate early intervention. Multivariate analysis showed that involvement of more than three venous sinuses, a platelet count < 225 × 10^9^/L, serum sodium < 139 mmol/L, seizures, oral contraceptive use, and papilledema were independently associated with an increased risk of DCP [56]. Based on these predictors, the authors developed a 10-point risk score, in which patients with a score ≥ 5 had at least a 50% probability of clinical deterioration. This CVT-DCP score provides a practical tool for early risk stratification, closer monitoring, and timely therapeutic escalation [56].

Also in 2018, Barboza MA et al. proposed the CVT grading scale as previously described in Disease Severity assessments [42]. Patients were retrospectively recruited from two tertiary care teaching centers in Mexico City. The CVT grading scale assigns weighted points to five independent predictors: parenchymal lesion size > 6 cm (3 points), bilateral Babinski signs (3 points), male sex (2 points), parenchymal hemorrhage (2 points), and level of consciousness impairment (3 points). The CVT grading scale with a total of 13 points demonstrated an accuracy of 91.6% in predicting 30-day mortality and 85.3% in predicting poor functional outcome defined as a mRS > 2.

The IN-REvASC score was proposed by Klein P et al. in 2022 and developed based on data from the Anticoagulation in the Treatment of Cerebral Venous Thrombosis (ACTION-CVT) cohort, which retrospectively included patients from the United States, Europe, and New Zealand [57]. It incorporates predictors including active cancer, age > 50 years, creatinine > 1 mg/dL, Black race, encephalopathy or coma, intracranial hemorrhage, hemoglobin < 12 g/dL, higher NIHSS scores, and substance use, yielding a total score of 35 points. The derived IN-REvASC score outperformed the ISCVT risk score in predicting poor outcomes at 90-day follow-up as well as mortality.

The Chinese CVT Outcome Score was proposed by Li M et al. [47] in 2023 and validated using variables tailored to the Chinese population. This score incorporates age > 27.5 years, NSE > 16.5 ng/mL, diastolic blood pressure > 79.5 mmHg, and an NLR > 6.6, yielding a total score of 7 points. A score > 3 demonstrated good discriminative ability for unfavorable outcomes at 6-month follow-up, highlighting the value of population-specific covariates in prognostic assessment.

The SI_2_NCAL_2_C score, proposed by Lindgren E et al. in 2023 [58], was developed based on data from patients in Europe, Australia, the Middle East, and Latin America. It incorporates the following components: the absence of female-sex-specific risk factors, intracerebral hemorrhage, infection in the central nervous system, neurological focal deficits, coma, age, a lower level of hemoglobin (g/L) at admission, a higher level of glucose (mmol/L) at admission, and cancer. The SI_2_NCAL_2_C score demonstrated acceptable to good performance in predicting poor outcomes at 90 days, as well as 30-day and 1-year mortality, in an international external validation using the ACTION-CVT cohort [59].

A summary of the prognostic scoring systems developed for patients with CVT is presented in Table 4. The ISCVT risk score is the most classic and widely used prognostic scale for CVT; however, it does not incorporate blood biomarkers or imaging metrics. The CVT-DCP score is specifically designed to predict DCP in CVT patients, but it was developed from a single-center cohort and lacks external validation. The Chinese CVT Outcome Score, derived from a Chinese cohort, may be more suitable for prognostic assessment in Chinese patients with limited external validation outside China. In contrast, the SI_2_NCAL_2_C and IN-REvASC scores were primarily derived from cohorts in Europe, North and South America, and Oceania, and may therefore be more applicable to these populations. However, both scores rely on formula-based risk estimation, which may limit its feasibility for routine bedside use.

### 4.5. Future Directions of the Scoring Systems

Despite the growing number of clinical and imaging-based scoring systems developed for CVT, several important limitations remain. First, substantial heterogeneity exists among available studies in terms of study design, patient populations, imaging protocols, and outcome definitions. This heterogeneity limits the direct comparability of different scoring systems and may affect their generalizability across diverse clinical settings. Second, many currently available models were derived from retrospective cohorts or single-center studies with relatively small sample sizes. Several scoring systems lack robust prospective validation or external validation across different populations. In particular, the predictive performance of some models may vary depending on geographic region, ethnicity, and underlying etiological factors. Large multicenter prospective cohorts are needed to validate existing scoring systems and compare their predictive performance. Third, most existing scoring systems focus primarily on either imaging findings, clinical severity, or prognostic indicators alone. Few models integrate multidimensional information, including imaging, clinical features, and circulating biomarkers. Future scoring systems should therefore aim to incorporate multimodal data to better reflect the complex pathophysiology of CVT.

## 5. Novel Therapeutic Strategies

Although anticoagulation remains the cornerstone of therapy for CVT, limitations of vitamin K antagonists and growing insights into disease mechanisms have prompted the development of alternative and adjunctive treatment strategies. Recent years have seen the emergence of novel approaches, including direct oral anticoagulants, corticosteroids, natural-origin agents, immunomodulatory therapy, endovascular treatment, optic nerve sheath fenestration and rDMS. The following sections summarize current evidence for these evolving therapeutic options and their potential roles in CVT management.

### 5.1. Direct Oral Anticoagulants

Anticoagulation remains the cornerstone of therapy for CVT. Patients are typically treated with heparin during the acute phase and subsequently transitioned to oral warfarin. Over the last decade, evidence supporting the use of direct oral anticoagulants (DOACs) in CVT has expanded substantially with the support from several randomized controlled trials (RCTs) and large real-world cohort studies. The Clinical Trial Comparing Efficacy and Safety of Dabigatran Etexilate with Warfarin in Patients with Cerebral Venous and Dural Sinus Thrombosis (RE-SPECT CVT) trial was the first randomized controlled trial (RCT) to compare dabigatran with warfarin in patients with CVT. The trial demonstrated that dabigatran is non-inferior to warfarin for the prevention of recurrent venous thromboembolism and exhibits a comparable safety profile, with similar rates of major bleeding [60]. The Dabigatran Etexilate Versus Warfarin in CVT in Chinese Patients (CHOICE-CVT) trial, an exploratory, single-center, open-label, RCT study in Chinese patients with CVT, compared dabigatran with warfarin following initial low-molecular-weight heparin (LMWH) therapy. The results were consistent with those of the RE-SPECT CVT, supporting dabigatran as a safe and effective anticoagulant alternative to warfarin in CVT [61].

Subsequently, several RCTs further expanded the evidence base. The Study of Rivaroxaban for Cerebral Venous thrombosis (SECRET) trial, an open-label, RCT study, compared rivaroxaban with standard anticoagulation in patients with CVT. At 6 months of follow-up, no recurrent venous thromboembolism occurred in either group, and the majority of patients achieved favorable functional outcomes. Venous recanalization rates were similar, and no major bleeding was observed in the rivaroxaban arm. These results support rivaroxaban as a safe and effective alternative to standard anticoagulation in CVT [62]. The Rivaroxaban Versus Warfarin for the Treatment of Cerebral Venous Thrombosis (RWCVT) trial was an open-label, RCT study conducted at three centers in Syria, comparing rivaroxaban with warfarin after initial LMWH in patients with CVT. Over 6 months of follow-up, there were no significant differences between the rivaroxaban and warfarin groups in functional outcomes, CVT recurrence, all-cause mortality and overall safety profiles. These findings are consistent with the results of the SECRET trial and confirm that rivaroxaban has comparable efficacy and safety to warfarin, supporting its use as an alternative oral anticoagulant in CVT [63].

Only a few case series have reported the use of apixaban in CVT, and no dedicated RCT studies have been conducted. A high proportion of partial or complete recanalization was achieved with apixaban treatment, and none of the case series reported any adverse events, including bleeding complications [64,65,66]. Edoxaban has also been explored as an alternative anticoagulant for CVT, but evidence remains limited. To date, no large RCTs have evaluated edoxaban in CVT. Existing data come primarily from case reports and small observational series, which suggest that edoxaban may achieve successful venous recanalization with a favorable safety profile in selected patients [67]. While these preliminary findings are encouraging, robust prospective studies are required to establish its efficacy and safety relative to warfarin or other direct oral anticoagulants.

Beyond RCTs, large real-world cohorts provide complementary evidence. The multinational retrospective ACTION-CVT study and the prospective Direct Oral Anticoagulants versus Vitamin K Antagonists for Cerebral Venous Thrombosis (DOAC-CVT) phase-IV cohort collectively indicate that DOACs achieve similar rates of functional recovery and venous recanalization compared with warfarin, with no increase in recurrent thrombosis and potentially lower rates of major bleeding [68]. A recent meta-analysis incorporating real-world cohorts confirmed that DOACs are comparable to warfarin in both effectiveness and safety, supporting their growing adoption in clinical practice [69].

CVT in children is rare but associated with significant morbidity. Standard anticoagulation typically involves LMWH or warfarin, with dosing carefully adjusted for age and weight. Emerging evidence suggests that DOACs, such as rivaroxaban and dabigatran, may be a feasible alternative in pediatric CVT. Although no large RCTs specifically in pediatric CVT have been conducted, data from pediatric venous thromboembolism studies, along with case reports and small cohort studies, indicate that DOACs can achieve effective recanalization with a favorable safety profile. Current guidelines, including the 2023 American Society of Hematology recommendations, acknowledge DOACs as an option for children with stable CVT and low bleeding risk, while emphasizing the need for careful monitoring, weight-based dosing, and consideration of renal function. Further prospective studies are required to confirm long-term efficacy and safety in this population [70].

CVT during pregnancy is rare but associated with significant maternal morbidity and requires prompt anticoagulation. Current guidelines recommend LMWH as the first-line therapy throughout pregnancy, given its proven efficacy and established safety profile for both mother and fetus. Warfarin is contraindicated during the first trimester due to teratogenicity but may be considered postpartum. DOACs are not recommended in pregnant patients, as they cross the placenta and there is a lack of safety data in this population. Therefore, while DOACs are increasingly used in non-pregnant adults for CVT, LMWH remains the anticoagulant of choice during pregnancy, with careful monitoring and dose adjustment according to maternal weight and gestational age.

### 5.2. Corticosteroids

Preclinical and clinical studies indicate that inflammatory pathways, such as the NLRP3 inflammasome, are highly activated in severe CVT, and corticosteroids may exert potential protective effects against brain tissue injury by suppressing these inflammatory responses [71]. Theoretical benefits of corticosteroids may also derive from their ability to stabilize the blood–brain barrier and reduce vasogenic edema, that may be particularly relevant in severe CVT with intracranial hypertension and extensive parenchymal lesions [37]. However, analysis of the ISCVT cohort indicated that routine corticosteroid use was not associated with improved short-term or long-term prognosis. Patients without parenchymal lesions treated with steroids had worse prognosis than those treated without steroids [72].

Notably, a recent prospective cohort study demonstrated that short-term pulsed steroid therapy combined with standard anticoagulation was associated with a higher rate of favorable functional outcomes at 6 months of follow-up compared with anticoagulation alone in patients with severe CVT. This benefit was accompanied by reductions in serum and cerebrospinal fluid inflammatory markers, decreased intracranial pressure, and improved fundoscopic findings, without an increase in mortality or severe steroid-related adverse events [38]. Whether pulsed steroids should be routinely used in severe CVT requires further high-quality RCTs to establish definitive clinical guidance.

### 5.3. Natural-Origin Agents: Batroxobin and Escin

Several natural-origin agents have attracted attention as adjunctive therapies, particularly in Asian clinical practice. Batroxobin, a thrombin-like serine protease derived from snake venom, directly reduces plasma fibrinogen levels and indirectly enhances fibrinolysis. In 2018, Ding et al. [73] reported that patients receiving batroxobin had a higher venous recanalization rate than those in the control group, without an increased risk of intracranial hemorrhage. However, no significant differences in mRS scores were observed at discharge or at 6 months [73]. Subsequently, Lan D et al. demonstrated that batroxobin significantly inhibited adenosine diphosphate-induced platelet aggregation, increased D-dimer levels, decreased fibrinogen concentrations, and prolonged thrombin time and activated partial thromboplastin time, supporting its anticoagulant and profibrinolytic effects [74]. Moreover, recent evidence suggests that batroxobin may exert anti-inflammatory benefits in CVT, as reflected by reductions in systemic inflammatory indices such as the NLR, platelet-to-lymphocyte ratio, and systemic immune–inflammation index [75]. Larger multicenter RCTs are needed before broad recommendations can be made.

Escin, a saponin compound, has venotonic, anti-inflammatory, and anti-edematous properties. However, the efficacy of escin has only been demonstrated in preclinical models. In a rat model of CVT, escin was shown to preserve blood–brain barrier integrity, attenuate brain edema, and significantly improve motor performance and neurological deficits. Mechanistically, escin markedly inhibited activation of the NLRP3 inflammasome pathway, reduced caspase-1 cleavage and gasdermin D-mediated pyroptosis, and suppressed the release of proinflammatory cytokines such as IL-1β and IL-18. This preclinical study suggests that, beyond any hemodynamic or antithrombotic effects, escin may exert neuroprotective actions in CVT by targeting NLRP3 inflammasome-driven sterile inflammation, thereby linking molecular anti-inflammatory effects with functional recovery [76,77]. RCTs or well-designed prospective studies are needed to further evaluate the efficacy and safety of escin in patients with CVT.

### 5.4. Endovascular Treatment (EVT)

EVT, including catheter-directed thrombolysis, mechanical thrombectomy, and venous sinus stenting, has increasingly been used as a rescue option for patients with severe or refractory CVT. EVT is technically feasible and can achieve high rates of acute recanalization in selected cases according to systematic reviews and observational studies. A meta-analysis of case series and retrospective reports suggested that EVT may be safe and effective in severe CVT, with a pooled complete recanalization rate of approximately 62% and good functional outcome in 85% of treated patients [78].

However, a large retrospective propensity-matched multinational cohort across the USA, Italy, Switzerland, and New Zealand found no clear benefit of EVT on primary functional outcomes in CVT [79]. A nationwide retrospective Japanese cohort study likewise reported that EVT did not improve in-hospital mortality or modified Rankin Scale outcomes compared with standard treatment and failed to identify specific subgroups with clear benefit [80]. The only multicenter RCT to date, TO-ACT, failed to demonstrate a significant improvement in long-term functional outcomes with EVT over standard medical care in patients with severe CVT [81]. Recent large observational evidence from a multicenter nationwide cohort in China involving over 2700 CVT patients also supported that EVT was not associated with improved rates of good functional recovery or reduced in-hospital mortality compared with standard care [82]. International practice patterns demonstrate substantial clinician support for EVT in selected clinical scenarios characterized by neurological deterioration despite optimal medical therapy. However, significant global variability exists in its utilization and in the factors guiding decision-making, highlighting the lack of consensus in the absence of high-quality evidence.

Technical innovation in EVT for CVT is ongoing, including the development and evaluation of dedicated venous thrombectomy devices aimed at improving technical success and identifying phenotypes that may derive true functional benefit from early EVT. A recent RCT study suggests that the dedicated venous thrombectomy device Venous-TD does not increase procedural complications and can significantly improve complete recanalization rates in EVT for CVT (NCT05291585). Nevertheless, a multicenter phase III RCT is still warranted to confirm definitive efficacy and safety [83]. Another ongoing studies, the ESCORT trial (NCT06583889), is designed to evaluate the safety and technical feasibility of the EmboTrap device in patients with severe or refractory CVT. Collectively, these efforts may clarify whether dedicated venous thrombectomy devices contribute to favorable functional outcomes and which phenotypes truly benefit from early EVT.

### 5.5. Optic Nerve Sheath Fenestration (ONSF)

Visual impairment in CVT is primarily attributed to sustained elevation of intracranial pressure and secondary optic nerve sheath hypertension. ONSF has been employed as a surgical adjunct in selected patients with cerebral venous thrombosis who develop refractory intracranial hypertension accompanied by progressive visual deterioration, particularly when anticoagulation and intracranial pressure-lowering agents fail to arrest visual loss [84]. By creating a fenestration in the optic nerve sheath to allow for local cerebrospinal fluid egress, ONSF reduces perineural pressure and thereby alleviates papilledema and visual impairment.

Several case reports have described visual improvement following ONSF in patients with CVT who developed severe papilledema refractory to standard therapy [85,86]. A retrospective observational study involving 18 patients with CVT and progressive visual impairment showed that ONSF resulted in stabilization or improvement of visual acuity in approximately 80.6% of treated eyes at 1 week and 60.7% at long-term follow-up over 6 months, and papilledema resolved in all cases [84].

Despite these encouraging observational findings, the total number of reported cases remains limited, and the available evidence consists mainly of retrospective cohorts and isolated case reports. No large prospective RCTs have yet been conducted to define optimal indications, timing, or long-term outcomes of ONSF in CVT. Accordingly, although ONSF appears to be a potentially effective adjunctive intervention for vision preservation in carefully selected CVT patients, its role remains poorly defined, and further high-quality studies are required to establish standardized criteria and to compare its long-term benefits.

### 5.6. Rhythmic Deep-Brain Magnetic Stimulation (rDMS)

Neuromodulation has emerged as a neurorestorative concept in venous stroke. rDMS represents a second-generation evolution of repetitive transcranial magnetic stimulation (rTMS). To improve safety, rDMS employs pulse-train stimulation combined with a low-intensity magnetic field, thereby reducing the risk of excessive cortical excitation. In contrast to conventional rTMS, rDMS can modulate deeper brain structures and has been miniaturized to a compact, suitcase-sized device, rendering it portable and suitable for potential home-based use [87,88].

rDMS has been shown to improve motor performance, enhance angiogenesis, and attenuate apoptosis through activation of the JAK2/STAT3 signaling pathway in a rat model of superior sagittal sinus thrombosis [87]. However, the available evidence is currently limited to preclinical settings. This preclinical study demonstrated that rDMS targets post-thrombotic neural repair rather than thrombus resolution. However, its clinical efficacy in patients has not yet been verified, and further well-designed clinical trials are required to support its translational potential. Although other physical neuromodulatory approaches, such as transcranial direct current stimulation, transcranial ultrasound stimulation and remote ischemic conditioning, have not yet been explored in CVT, they may also represent promising adjunctive therapeutic strategies [89,90,91].

## 6. Conclusions

Recent advances have enhanced our understanding of the complex pathophysiology of CVT, which involves thrombus initiation, propagation, venous hypertension with blood–brain barrier disruption, and secondary parenchymal injury. Comprehensive clinical assessment, including diagnosis and differential diagnosis, disease severity scores, imaging-based metrics, and prognostic scoring systems, enables accurate evaluation and risk stratification. Emerging therapeutic strategies, including direct oral anticoagulants, corticosteroids for selected patients, natural-origin agents, immunomodulatory therapy, endovascular treatment, optic nerve sheath fenestration, and neuromodulation, provide novel and alternative options for the management of CVT. Integrating mechanistic insights with refined clinical assessment and novel treatments may optimize patient management and guide future research in CVT.

## Figures and Tables

**Table 1:** Comparison of pathological mechanisms, clinical manifestations, neuroimaging features, and biomarker profiles between CVT and CVSS, with an emphasis on differential diagnosis.

Features	CVT	CVSS
**Pathology**	Intraluminal thrombus formation	Non-thrombotic venous sinus narrowing
**Imaging appearance of intraluminal filling defects**	abrupt or irregular narrowing	Smooth or tapered narrowing
**Imaging markers of intracranial hypertension**	Less frequent	More frequent
**Parenchymal involvement**	Venous infarction and/or intracerebral hemorrhage	Rare
**Contrast-enhanced black-blood MRI**	Demonstrates intraluminal thrombus	Shows absence of intraluminal thrombus
**Biomarkers**	Elevated D-dimer, fibrinogen, and IL-6	Normal D-dimer, fibrinogen, and IL-6

**Table 2:** Tools for assessing disease severity in CVT. This table compares commonly used traditional neurological scales and CVT-specific severity scales in terms of their assessment of intracranial hypertension, focal neurological deficits, diffuse encephalopathy, and cavernous sinus syndrome.

Assessment Tools		Author (Year)	Intracranial Hypertension	Focal Neurological Deficits	Diffuse Encephalopathy	Cavernous Sinus Syndrome	CVT Severity Classification
**Traditional neurological scales**	GCS	Teasdale and Jennett (1974) [39]			√		
	NIHSS	Brott T et al. (1989) [40]		√			
	CSF pressure	Biousse V et al. (1999) [41]	√				
**CVT-specific scales**	CVT grading scale	Barboza MA et al. (2018) [42]	√	√	√	√	Mild: 0–10; severe: >10
	CVT severity scale	Li M et al. (2023) [43]		√			Mild: 0–2; moderate: 3–7; severe: 8–13

**Table 3:** Imaging scoring system for CVT, including venous occlusion, recanalization, and collateral grades.

Imaging Scoring Systems	Author (Year)	Grade	Definition
**Venous occlusion scores**	Zubkov AY et al. (2009) [48]	0–11	3 points: the SSS was divided into thirds, and 1 point was assigned to each third; 1 point: the straight sinus, vein of Galen, each internal cerebral vein, each transverse sinus, and each sigmoid sinus; The total score was calculated by summing the scores of each affected sinus.
	Wu Z et al. (2021) [50]	0–10	2 points: the SSS; 1 point: the inferior sagittal sinus, straight sinus, each transverse sinus, each sigmoid sinus, and each internal jugular vein; The total score was calculated by summing the scores of each affected sinus.
	Wang Z et al. (2023) [51]	0–30	0 point: absence of thrombus; 1 point: thrombus occupying ≤50% thrombus; 2 points: occupying >50% but <100%; 3 points: complete occlusion; The total score was calculated by weighting the transverse sinus, sigmoid sinus, and internal jugular vein scores according to the side-specific drainage ratio and summing them with the scores of the superior sagittal sinus, straight sinus, and torcular Herophili.
**Recanalization grades**	Qureshi AI et al. (2010) [52]	I	Partial recanalization in ≥1 previously occluded sinus.
		II	Complete recanalization of ≥1 previously occluded sinus, with persistent occlusion in other sinuses.
		IIA	Complete recanalization without residual flow defect.
		IIB	Complete recanalization with residual non-occlusive flow defect.
		III	Complete recanalization of all previously occluded sinuses.
	Aguiar De Sousa et al. (2020) [53]	0	Persistent occlusion of all previously thrombosed sinuses.
		1	Persistent occlusion in ≥1 sinus.
		1A	Persistent occlusion plus partial recanalization.
		1B	Persistent occlusion plus complete recanalization.
		2	No persistent occlusion.
		2A	Partial recanalization of all thrombosed sinuses.
		2B	Combination of partial and complete recanalization across sinuses.
		3	Complete recanalization of all previously thrombosed sinuses.
**Collateral circulation grades**	Qureshi AI et al. (2010) [52]	I	Collateral veins bypass the occluded segment of dural venous sinus but connect within the same sinus.
		II	Collateral veins bypass the occluded segment of dural venous sinus but connect with a different sinus.
		III	Collateral veins bypass the occluded segment of dural venous sinus but connect with a different circulation.
	Sheth SA et al. (2018) [54]	0	No venous drainage from affected region.
		1	Cortical venous drainage from affected region without anastomosis to a patent sinus.
		2	Cortical venous drainage from affected region with anastomosis into a patent sinus.

**Table 4:** This table summarizes the prognostic scoring systems developed for patients with cerebral venous thrombosis (CVT). For each score, the first author and year of publication, study design, recruitment setting, sample size, total score range, recommended cutoff value for predicting poor outcome, and individual score items are presented.

Prognostic Scores	Author (Year)	Study Design	Patient Recruitment	Sample Size (*n*)	Score Range	Cutoff for Poor Prognosis	Items
**ISCVT risk score**	Ferro JM et al. (2009) [55]	Prospective, multicenter	Europe, South America, North America, Australia, China, and India	624	0–9	≥3	Male, age > 37, mental status disturbance, intracranial hemorrhage, and deep venous thrombosis
**DCP prediction score**	Bushnaq SA et al. (2018) [56]	Retrospective, single-center	United States	147	0–10	≥5	Involvement of more than three venous sinuses, a platelet count < 225 × 10^9^/L, serum sodium < 139 mmol/L, seizures, oral contraceptive use, and papilledema
**CVT grading scale**	Barboza MA et al. (2018) [42]	Retrospective, multicenter	Mexico	467	0–13		Male, parenchymal lesion size > 6 cm, bilateral Babinski signs, parenchymal hemorrhage, and level of consciousness impairment
**IN-REvASC score**	Klein P et al. (2022) [57]	Retrospective, multicenter	United States, Europe, and New Zealand	554	0–35		Age > 50, Black race, creatinine > 1 mg/dL, encephalopathy or coma, intracranial hemorrhage, Hb < 12 g/dL, higher NIHSS, active cancer, and substance use
**Chinese CVT Outcome score**	Li M et al. (2023) [47]	Prospective, multicenter	China	170	0–7	>3	Age > 27.5, NSE > 16.5 ng/mL, diastolic blood pressure > 79.5 mmHg, and NLR > 6.6
**SI_2_NCAL_2_C score**	Lindgren E et al. (2023) [58]	Retrospective and prospective, multicenter	Europe, Australia, Middle East, and Latin America	1455			Age, female-specific risk factors, intracerebral hemorrhage, infection in the central nervous system, neurological focal deficit, coma, hemoglobin, blood glucose, cancer

## Data Availability

No new data were generated from this study.

## Abbreviations

ACTION-CVT	Anticoagulation in the Treatment of Cerebral Venous Thrombosis
BBB	blood–brain barrier
CHOICE-CVT	Dabigatran Etexilate Versus Warfarin in CVT in Chinese Patients
CSF	cerebrospinal fluid
CTV	computed tomography venography
CVSS	cerebral venous sinus stenosis
CVST	cerebral venous sinus thrombosis
CVT	cerebral venous thrombosis
DCP	delayed clinical progression
DFR	D-dimer-to-fibrinogen ratio
DOACs	direct oral anticoagulants
DOAC-CVT	Direct Oral Anticoagulants versus Vitamin K Antagonists for Cerebral Venous Thrombosis
DSA	digital subtraction angiography
EVT	endovascular treatment
GCS	Glasgow Coma Scale
Hs-CRP	high-sensitivity C-reactive protein
ICP	intracranial pressure
IL	interleukin
ISCVT	International Study on Cerebral Vein and Dural Sinus Thrombosis
LMWH	low-molecular-weight heparin
LPO	lipid peroxidation
MMP-9	matrix metalloproteinase-9
MRV	magnetic resonance venography
NETs	neutrophil extracellular traps
NIHSS	National Institutes of Health Stroke Scale
NLR	neutrophil-to-lymphocyte ratio
NLRP3	NOD-like receptor family pyrin domain-containing 3
NSE	neuron-specific enolase
ONSF	optic nerve sheath fenestration
PAI	plasminogen activator inhibitor
PF4	platelet factor 4
RCT	randomized controlled trial
rDMS	rhythmic deep-brain magnetic stimulation
RE-SPECT CVT	A Clinical Trial Comparing Efficacy and Safety of Dabigatran Etexilate with Warfarin in Patients with Cerebral Venous and Dural Sinus Thrombosis
ROS	reactive oxygen species
RWCVT	Rivaroxaban Versus Warfarin for the Treatment of Cerebral Venous Thrombosis
rTMS	repetitive transcranial magnetic stimulation
SECRET	Study of Rivaroxaban for Cerebral Venous thrombosis
SSS	superior sagittal sinus
tPA	tissue-type plasminogen activator
uPA	urokinase-type plasminogen activator
VCS	venous collateral scale
VOIS	Venous Occlusion Image Score
ZO-1	zonula occludens-1
